# Efficacy and safety of acupuncture for vocal nodules: A systematic review and meta-analysis with trial sequential analysis

**DOI:** 10.1371/journal.pone.0288252

**Published:** 2023-11-03

**Authors:** Zhixian Xiao, Qinwei Fu, Xiaocui Long, Li Zhou, Ruijing Zhu, Qianlin Peng, Xingbi Xie, Yiru Liao

**Affiliations:** 1 Traditional Chinese Medicine Hospital of Meishan, Meishan, P. R. China; 2 Hospital of Chengdu University of Traditional Chinese Medicine, Chengdu University of Traditional Chinese Medicine, Chengdu, P. R. China; 3 Sichuan Second Hospital of Traditional Chinese Medicine, Chengdu, P. R. China; Beijing University of Chinese Medicine, CHINA

## Abstract

In this study, we aim to evaluate the efficacy and safety of acupuncture for vocal nodules, concerning qualitive overall efficacy and quantitative improvement on quality of voice. Four English and four Chinese databases were searched up to December 10^th^, 2022. Risk of bias among the included trials were evaluated by the Cochrane ROB tool. Systematic reviews and meta-analyses were conducted based on the Cochrane systematic review method by using RevMan 5.4 Software, and trial sequential analyses were performed by TSA 0.9. Meta-influence analyses, subgroup-analyses, meta-regression, and evaluation of publication bias were performed for exploration of heterogeneity by Stata V.14. Quality of the results was accessed through the GRADE-pro GDT. Cluster analyses and correlation coefficient were performed by R 4.1.3. Finally, 15 trials involving 1,888 participants were included. Results showed that compared with western medicine alone or Chinese herbal medicine alone, acupuncture alone yielded significantly (p<0.05) higher clinical effective rate and more improvement on scores of voice analyses. However, reduction on scores of grade, roughness, and breathiness and voice handicap index during follow-ups, and results of clinical effective rate suggested that acupuncture was inferior to voice training. In addition, meta-regression and sub-group analyses firstly revealed advanced efficacies of acupuncture when performed with local and remote acupoints, compared with local acupoints only. Acupuncture specified adverse event was denied in six trials while it was not mentioned in other nine trials. Results of cluster analyses and correlation coefficient showed that *Kai yin yi hao* and He gu (LI-4) were the most frequently applied matching-acupoints in trials. In conclusion, compared with western medicine (level of evidence: low ⨁⨁◯◯, GRADE C) and Chinese herbal medicine (level of evidence: moderate ⨁⨁⨁◯, GRADE B), acupuncture is safe and of better efficacy for patients with vocal nodules, while there is also need for RCTs with improvements on designing and interventions in experimental and controls.

## Introduction

Vocal nodules (VN) belong to the most common benign vocal cord disease, and is characterized by bilateral nodular protrusions in the anterior 1/3 of the vocal cords and voice disorder [1]. Researches have suggested potential associations between VN and phonetic trauma/abuse, while their exact etiopathogeneses remain unknown [2, 3]. Due to hoarseness, vocal fatigue and pharyngeal discomfort, VN decreases patients’ quality of life, physiologically and socially [4]. According to a Korean epidemiological report, the incidence of VN was 0.99%-1.72%, while it was more frequent in male children and adult female, which was same as a Turkish survey (diagnosed in 30.3%, 187 cases, of school age children) [5, 6].

Formally, therapies for VN are divided into surgical and non-surgical approaches, and specific superiority or inferiority concerning any of them were still not evaluated in a Cochrane systematic review [7]. However, clinically, non-surgical therapies are more selected and better preferred by medical staff and patients, which may owe to its non-invasive nature. Among them, evidence showed that voice training (VT) is beneficial in improving voice quality, while it was not popular in most of the world due to insufficient (or without) speech pathology specialty and limited medical resources [8]. In addition to VT and other non-surgical therapies, some VN patients have been turning to complementary and alternative therapies, acupuncture and Chinese herbal medicine (CHM) especially, for better efficacy, longer duration and fewer costs.

In traditional Chinese medicine (TCM), VN lies in *Qi* stagnation, phlegm, fluid retention, and blood stasis, which are resulted from unbalanced visceral function, and gather in vocal cord [9]. Originating about three thousand years ago, acupuncture could regulate *Yin*, *Yang*, and visceral function based on the principles of meridians and acupoints of TCM, as well as circulate qi and blood [10, 11]. As a frequently used adjuvant, trials suggested potential benefits of acupuncture for benign nodular/hyperplasia diseases, such as benign prostatic hyperplasia, hyperplasia of mammary glands, and thyroid nodule [12–14].

However, no systematic review has been published to explore efficacy and safety of acupuncture for VN. The aim of this systematic review and meta-analysis is to fill the vacancy above with rigorous design and comprehensive analyses, and incorporate new evidence about acupuncture for VN. In addition, potential variations between studies, quality of outcomes and strength of evidence recommendations were also measured for better clinical application.

## Material and methods

### Protocol and registration

This systematic review was registered in PROSPERO with the registration number CRD42022350916 (available from https://www.crd.york.ac.uk/prospero/display_record.php?RecordID=350916).

### Search strategy

Four English databases, including PubMed, Embase, Cochrane Library, Web of Science, and four Chinese databases, including Chinese Biomedical Literature Database, VIP Database for Chinese Technical Periodicals, China National Knowledge Infrastructure, and Wanfang Database, were searched from inception until December 10^th^, 2022. The PRISMA agreement was followed in decision of search strategy and inclusion criteria [15]. Two subsets of terms were searched with the term ‘AND’, including the experimental intervention (‘acupuncture’, ‘needle’, ‘electro-acupuncture’, ‘electropuncture’, ‘electroacupuncture’, ‘acusector’, and ‘acupoint’) and terms of the disease (‘vocal nodule’, ‘behavioral voice disorder’, ‘voice disorder’, ‘voice’, ‘vocal cord’, ‘hoarseness’, ‘speech disorder’) (S1 File). Two authors processed the searches independently, and we also searched the references of the original and review studies manually for trials.

### Inclusion criteria

1) Trials in which participants were diagnosed with vocal nodules by laryngoscopy, and there was no limitation on type of laryngoscopy and vocal nodules;

2) Prospective randomized controlled trials (RCTs).

3) Trials in which acupuncture (manual or electro type) was applied as the only therapy in experimental groups. The participants in control groups received sham acupuncture, blank (wait-list) controls, western medicine (WM), TCM, VT, etc.. There was no restriction on duration, frequency, acupoint, or stimulation of acupuncture, while trials with other acupoint-based therapy (e.g., acupressure, moxibustion, acupoint injection, catgut embedding) designed as the control were excluded.

4) Primary outcomes included clinical effective rate (CER). Secondary outcomes included scales on objective sound quality and scores of subjective symptoms.

5) Trials published in Chinese or English.

### Study selection and data extraction

Two reviewers (RZ and QF) searched the online databases listed above and recorded the titles and abstracts of all the articles. Two evaluators (XL and YL) assessed the eligibility of these articles and made decisions on every research (inclusion or exclusion) independently. If they did not reach the same decision, the concerned articles were discussed with a fourth reviewer (ZX). Two reviewers (QP, and XX) extracted data independently from each study. Differences of extracted data were solved after discussion with a third reviewer (LZ).

### Quality assessment

Quality assessment of all the trials included in this review was independently evaluated by three reviewers (QF, XL and RZ) using the Cochrane Collaboration risk of bias tool by RevMan 5.4 software. Any disagreement was resolved by discussions with a fourth reviewer (ZX or LZ).

### Statistical analysis

Statistical analyses were measured with RevMan V.5.4, Stata V.14 and TSA 0.9 software. Effect sizes were determined as weighted mean difference (WMD) or standard mean difference for continuous outcomes, and risk ratio (RR) for binary outcomes with their 95% confidence intervals (CI). The Q and I^2^ test statistics were conducted to examine heterogeneity, with I^2^>50% indicating significant heterogeneity, with fixed or random-effects model applied, with P < .05 indicating significant differences for effect sizes. If the heterogeneity was still obvious (I^2^>50%) and more than two trials were included, then meta-influence analysis (for sensitivity analysis) was conducted [16].

Further, meta-regression and regression-based sub-group analyses for the primary outcome were performed to identify potential variables leading to high heterogeneity with interpretation. Exploration of publication bias by Egger’s tests were planned for the primary outcome, together with trim and fill test for further identification of the stability [16]. P < .05 indicates significant differences for meta-regression, and p<0.1 for Egger’s test.

For the primary outcome, trial sequential analyses were performed by TSA 0.9 with type I error α = 0.05 and type II error β = 0.1, aiming at examining and minimizing the impact of type 1 errors due to sparse data and repeated significance testing following updates with new trials [17]. We also conducted penalized test for further verification. Strength of evidence recommendations for effect estimation in each outcome was evaluated by The Grading of Recommendations Assessment, Development and Evaluation system (GRADE) pro GDT [18]. In addition, cluster analyses and correlation coefficient of the variables were performed concerning acupoints applied among the included trials by R 4.1.3.

## Results

### Study inclusion

**Fig 1:**
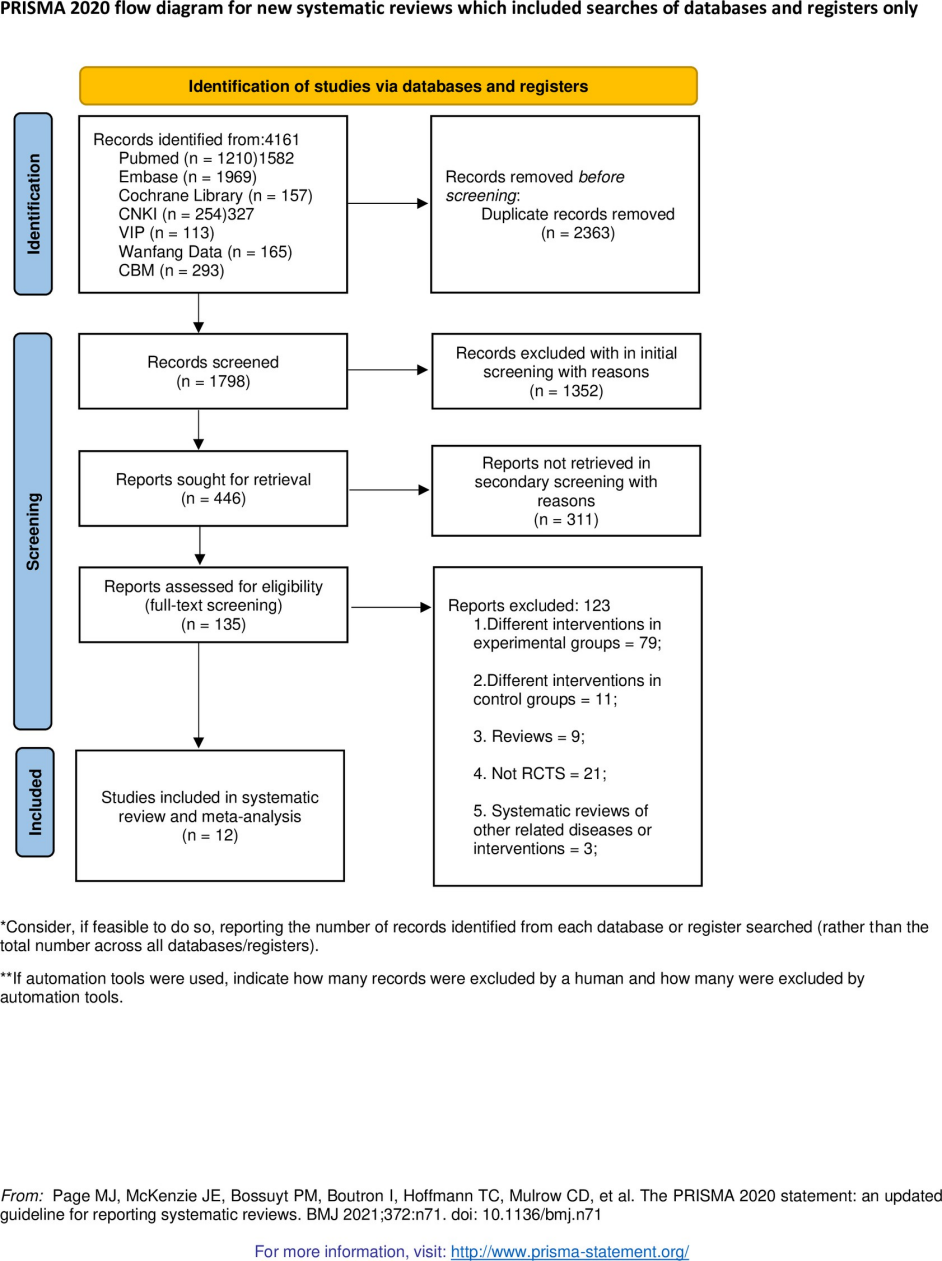
The PRISMA flow diagram of the study selection process.

Originally, 4161 studies were retrieved from the seven databases, of which 2363 were removed due to duplication, and 1352 studies were eliminated according to titles and abstracts. The remaining 446 studies were downloaded for further consideration, and among which 311 studies were excluded with reasons. Finally, 15 trials from 12 studies (three three-arm studies were recombined to six trials for comparison) were included (Fig 1) [19–30].

### Study characteristics

All of the 15 trials were conducted in China and published in Chinese, with the range of publish years from 1999 to 2022 [19–30]. Specifically, 1118 participants aging from 12 to 65 with VN from 20 days to 6 years were involved. As for experimental intervention, acupuncture alone was performed in all of the groups, with WM alone applied in six trials, CHM alone applied in eight trials, and VT alone in one trial. Though some of the specific prescriptions of the acupoints and TCM decoction applied were different among the included trials, their principles and theories showed similarity according to the theory of TCM. detailed characteristics of the included trials are listed in S1 Table. In addition, two specific acupoints, *Kai yin yi hao* and *Sang yin point*, were applied among the trials. Located on neck, the two acupoints are widely selected for improving quality of voice during acupuncture concerning VN. Location of *Kai yin yi hao* and *Sang yin point*, and summary of acupoints applied and frequency in the included trials are listed in S2 Table.

### Assessment of quality and bias

According to the Cochrane Handbook and risk of bias tool [16], the randomization methods were described clearly and appropriately in 10 trials [20–25, 29, 30], but were not detailed in the other trials. The manners of allocation concealment, blinding of outcome assessment, and selective reporting were assessed as unclear bias in all the trials due to insufficient reporting in full-text. The situation was same concerning blinding of participants and personnel, but we evaluated it as high bias in all the trials because it was impossible to perform the blinding between acupuncture and the controls (WM, CHM, and VT), obviously. In addition, all included trials were better reported in terms of completeness of outcome data, which were rated as low risk (S1 and S2 Figs).

### Pooled results of acupuncture for patients with vocal nodules

#### Effects of acupuncture vs. WM

**Fig 2:**
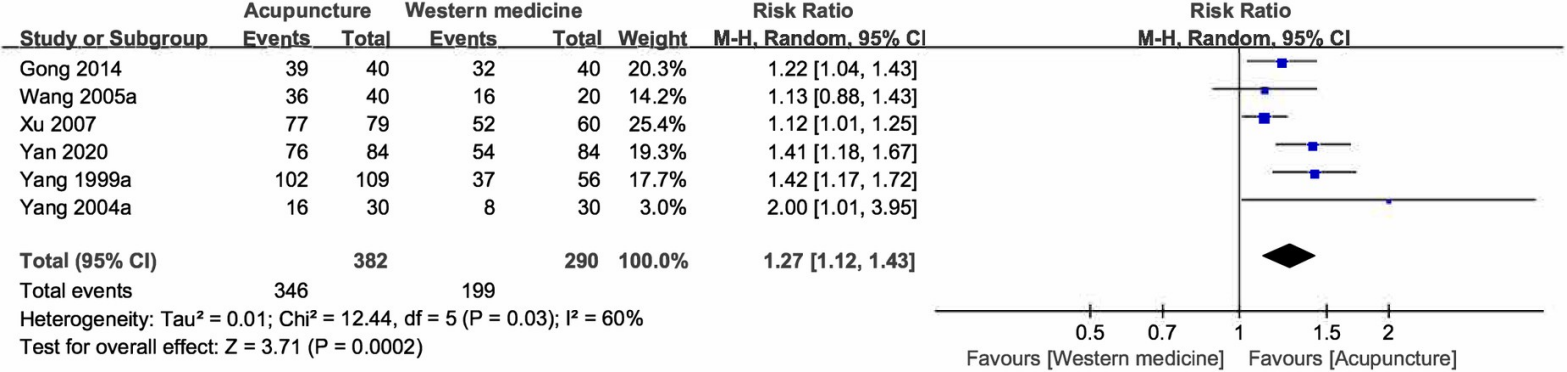
Forest plot for acupuncture vs. western medicine on clinical effective rate.

Pooled results favored acupuncture groups with significantly (p < 0.05) higher CER (RR = 1.27, P for RR = 0.002, 95%CI:1.12–1.43, I^2^ = 60%) [19, 23, 26–29] (Table 1 and Fig 2). Heterogeneity was still high (I^2^ > 50%) with random-effects model applied, and without extreme trial evaluated in meta-influence analysis (S3 Fig). Furtherly, meta-regression and sub-group analysis according to variations of age, gender, duration of disease, frequency of treatment, course of treatment, selection of acupoints, and report of *Deqi* were designed. Results showed that the heterogeneity was reduced to acceptable level (I^2^ < 50%) with statistical significance for meta-regression (tau^2^ = 0, I^2resid^ = 0%, Adjusted R^2^ = 100%, *P* = 0.04) when were sub-grouped based on selection of acupoints (local acupoints only, or local and remote acupoints) (Table 2 and S4 Fig).

**Table 1:** Summary of pooled results on acupuncture vs. control for patients with vocal nodules.

Control	Outcomes	No. of trials	Participants	Effect size	95% CI	P value of effect size	I^2^ value
**Western medicine**	Clinical effective rate	6	672	1.27 (RR)	1.12, 1.43	0.002	60%
**Chinese herbal medicine**	Clinical effective rate	6	455	1.16 (RR)	1.06, 1.26	0.001	0%
	Reduction on symptom scores	2	97	3.92 (WMD)	2.65, 5.19	< 0.001	0%
	Improvement on scores of voice analyses	2	90	3.70 (WMD)	2.26, 5.15	< 0.001	0%
**Voice training**	Clinical effective rate	1	80	0.78 (RR)	0.59, 1.04	0.09	NA
	Reduction on scores of GRB (one month after treatment)	1	80	-0.75 (WMD)	-1.13, -0.37	< 0.001	NA
	Reduction on scores of GRB (two months after treatment)	1	80	-0.65 (WMD)	-1.41, 0.11	0.09	NA
	Reduction on scores of VHI (one month after treatment)	1	80	-5.67 (WMD)	-9.00, -2.34	< 0.001	NA
	Reduction on scores of VHI (two months after treatment)	1	80	-3.75 (WMD)	-9.14, 1.64	0.17	NA

CI: Confidence interval; RR: Risk ratio; WMD: Weighted mean difference; GRB: A tool for evaluation of hoarseness, including Grade (overall grade of hoarseness), Roughness (roughness of voice), and Breathiness (breathiness of voice) by doctors; VHI: Voice handicap indexo; NA: Not applicable.

**Table 2:** Summary of meta-regression and subgroup-analyses for clinical effective rate (acupuncture vs. western medicine).

Characteristics	Meta-regression				Subgroup analysis			
	tau^2^	I^2^_resid_	Adjusted R^2^	P value	No. of trials	RR (95% CI)	P value of effect size	I^2^
**Age, years** *								
** 30±2.5**	0.01	44.83%	-37.39%	0.33	2	1.25 (0.96, 1.62)	0.098	82.5%
** 35±2.5**				0.26	2	1.19 (1.04, 1.36)	0.012	0%
** 40±2.5**	Ref				2	1.46 (1.17, 1.81)	0.001	8.5%
**Gender (proportion of male/female), rank**								
** The top 50% of trials (higher proportions)**	0.01	58.13%	-49.27%	0.74	3	1.29 (1.07, 1.57)	0.009	78.2%
** The final 50% of trials (lower proportions)**					3	1.23 (1.01, 1.50)	0.043	38.4%
**Duration of disease, year** *								
** < 1**	0.01	43.45%	21.39%	0.27	2	1.46 (1.17, 1.81)	0.001	8.5%
** ≥ 1**					3	1.21 (1.02, 1.43)	0.028	63.7%
**Frequency of treatment (acupuncture)** *								
** qd**	0	30.55%	100%	0.18	3	1.20 (1.01, 1.43)	0.036	56%
** Fewer than qd**					3	1.34 (1.17, 1.53)	<0.001	25.6%
**Course of treatment (acupuncture), month** *								
** <1**	0.01	58.13%	-49.27%	0.74	3	1.29 (1.07, 1.57)	0.009	78.2%
** 1**					3	1.23 (1.01, 1.50)	0.043	38.4%
**Selection of acupoints**								
** Local acupoints only**	0	0%	100%	0.04	3	1.15 (1.06, 1.25)	0.001	0%
** Local and remote acupoints**					3	1.43 (1.26, 1.62)	<0.001	0%
**Report of *Deqi* (obtaining qi in traditional Chinese medicine) during acupuncture**								
** Yes**	0.01	55.36%	-72.28%	0.93	3	1.26 (1.11, 1.43)	<0.001	26.2%
** Not reported**					3	1.34 (0.99, 1.80)	0.06	80%

RR: Risk Ratio; qd: Once per day; Ref: Reference.

*The values were mean or median.

#### Effects of acupuncture vs. CHM

**Fig 3:**
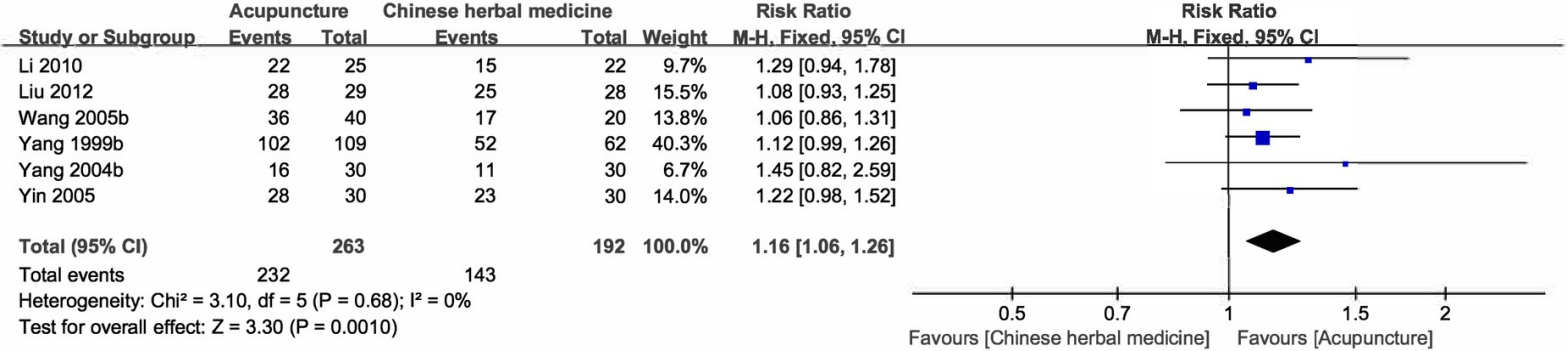
Forest plot for acupuncture vs. Chinese herbal medicine on clinical effective rate.

Compared with CHM, results of systematic reviews favored acupuncture groups with significantly (p < 0.05) higher CER (RR = 1.16, P for RR = 0.001, 95%CI:1.06–1.26, *I*^*2*^ = 0%) (Table 1 and Fig 3) [20, 21, 23, 28–30]. Findings of meta-analyses also showed that acupuncture could reduce symptom scores (WMD = 3.92, P for WMD < 0.001, 95%CI:2.65–5.19, *I*^*2*^ = 0%) [20, 24] (Table 1 and S5 Fig), and improve scores of voice analyses (WMD = 3.70, P for WMD <0.001, 95%CI:2.26–5.15, *I*^*2*^ = 0%) more, significantly (p < 0.05) (Table 1 and S6 Fig) [24, 25].

#### Effects of acupuncture vs. VT

However, results of one trial suggested that acupuncture was inferior to VT concerning lower CER (RR = 0.78, P for RR = 0.09, 95%CI:0.59–1.04, *I*^*2*^ = NA) (Table 1 and S7 Fig) [22]. Similarly, during follow-ups, results also suggested fewer reduction on scores of grade, roughness, and breathiness (GRB) in one month after treatment (WMD = -0.75, P for WMD < 0.001, 95%CI:-1.13 - -0.37, *I*^*2*^ = NA), scores of GRB in two months after treatment (WMD = -0.65, P for WMD = 0.09, 95%CI:-1.41–0.11, *I*^*2*^ = NA), scores of voice handicap index (VHI) in one month after treatment (WMD = -5.67, P for WMD < 0.001, 95%CI:-9.00 - -2.34, *I*^*2*^ = NA), and scores of VHI in two months after treatment (WMD = -3.75, P for WMD = 0.17, 95%CI:-9.14–1.64, *I*^*2*^ = 0%) for the experimental compared with VT [22] (Table 1 and S8–S11 Figs).

#### Meta-regression based sub-group analyses of the effects

For the outcomes of CER (acupuncture vs. WM and acupuncture vs. CHM), sub-group analyses based on meta-regression were performed to explore characteristics which might influence sizes of effect and heterogeneities, potentially. Sub-group analyses were also designed based on variations of age, gender, duration of disease, frequency of treatment, course of treatment, selection of acupoints, and report of *Deqi*. Results of sub-group analyses revealed some common findings concerning the two comparisons (Table 2 and S3 Table). Firstly, groups with older ages (40 ± 2.5, years) yielded comparatively higher RR than younger ages (30 ± 2.5, years and 35 ± 2.5, years). Secondly, results favored the experimental with comparatively higher RR when more proportions of male patients were included. Thirdly, as for duration of disease, results favored the experimental with comparatively higher RR when the time was shorter than one year, compared with those were not shorter than one year. Fourthly, as for course of treatment, results favored the experimental with comparatively higher RR when it was shorter than one month, compared with those were one month. Finally, groups with local and remote acupoints applied yielded comparatively higher RR than those with local acupoints applied only.

### Trial sequential analysis and penalized test

**Fig 4:**
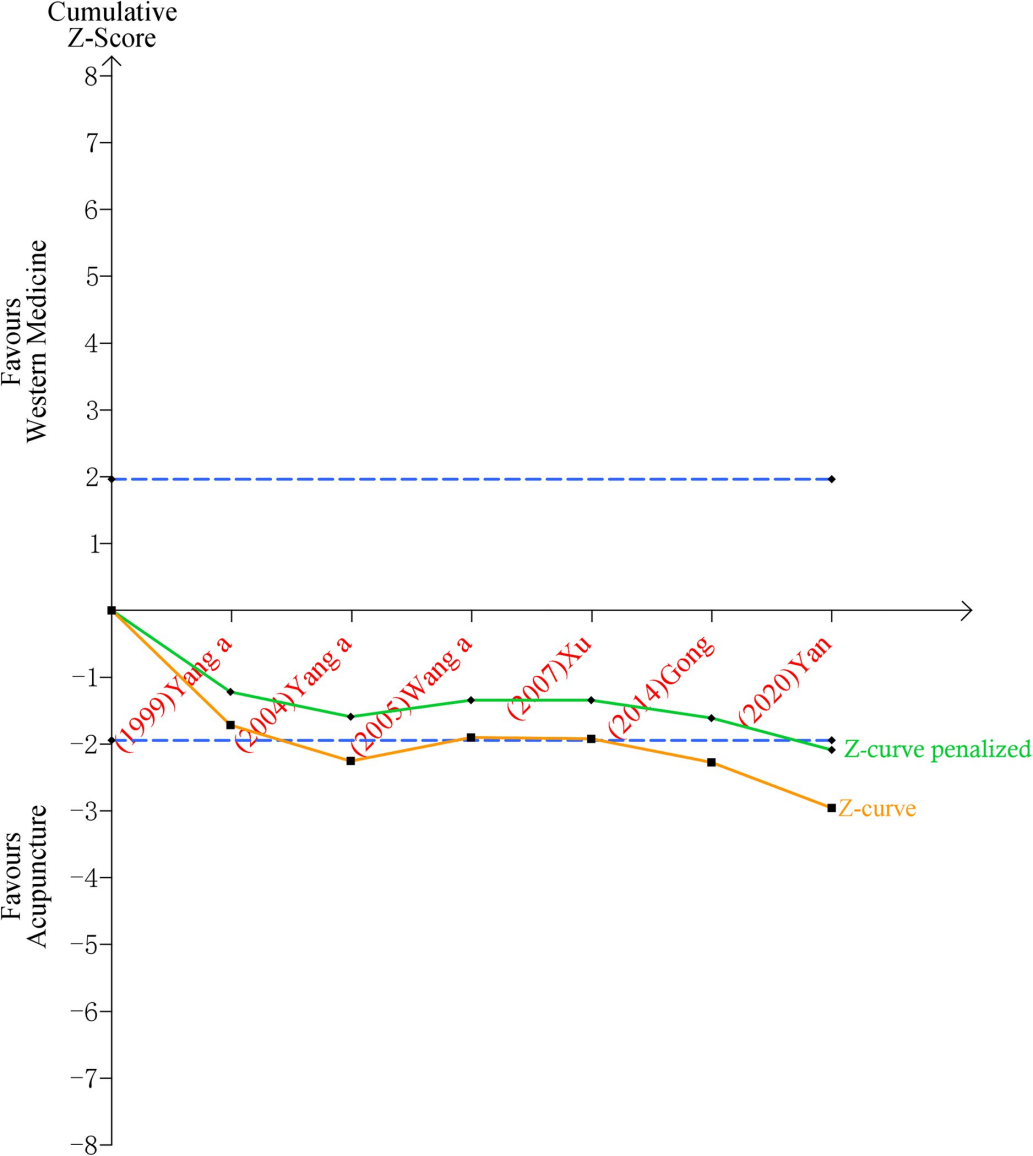
Trial sequential analysis and penalized test for acupuncture vs. western medicine on clinical effective rate. Notes: RIS: Required information size. This picture showed that 1) Z-curve didn’t cross the RIS, indicating that the number of included studies has not reached the amount required for meta-analysis; 2) Z-curve crossed the conventional boundary of benefit (z = 1.96), indicating that the difference of clinical effective rate between acupuncture group versus western medicine group was statistically significant, excluding the possibility of false positive; 3) Z-curve crossed trial sequential monitoring boundary for benefit, which indicated that acupuncture was not superior to western medicine.

Trial sequential analysis and penalized tests were conducted for CER of acupuncture vs. WM and acupuncture vs. CHM. For the two outcomes, results favored exclusion of the possibility of false positive before and after penalized test (Fig 4, S12 Fig). However, the results need to be interpreted with caution because of the lack of inclusion (≤10 trials), more inclusion was needed to reach enough superiority and to meet the required information size for them.

### Adverse event reported in trials

Among the included trials, six of them reported that there was no acupuncture specific adverse event [19, 20, 22, 24, 25, 30], and it was not mentioned in other nine trials [21, 23, 26–29].

### Publication bias and trim and fill test

Egger’s test was performed in two comparisons, and publication bias was not detected in both of them (S13 and S14 Figs). Trim and fill test showed that statistical significances of the two comparisons were not reversed after certain number of missing studies filled, indicating the stability of them (S15 and S16 Figs).

### Levels of evidence

As for acupuncture vs. CHM, levels of evidence were suggested as moderate (⨁⨁⨁◯, GRADE B) for all of the three comparisons (S4 Table). However, levels of evidence for all comparisons concerning acupuncture vs. WM and acupuncture vs. VT were low (⨁⨁◯◯, GRADE C) (S4 Table).

### Cluster analyses and correlation coefficient of the variables

**Fig 5:**
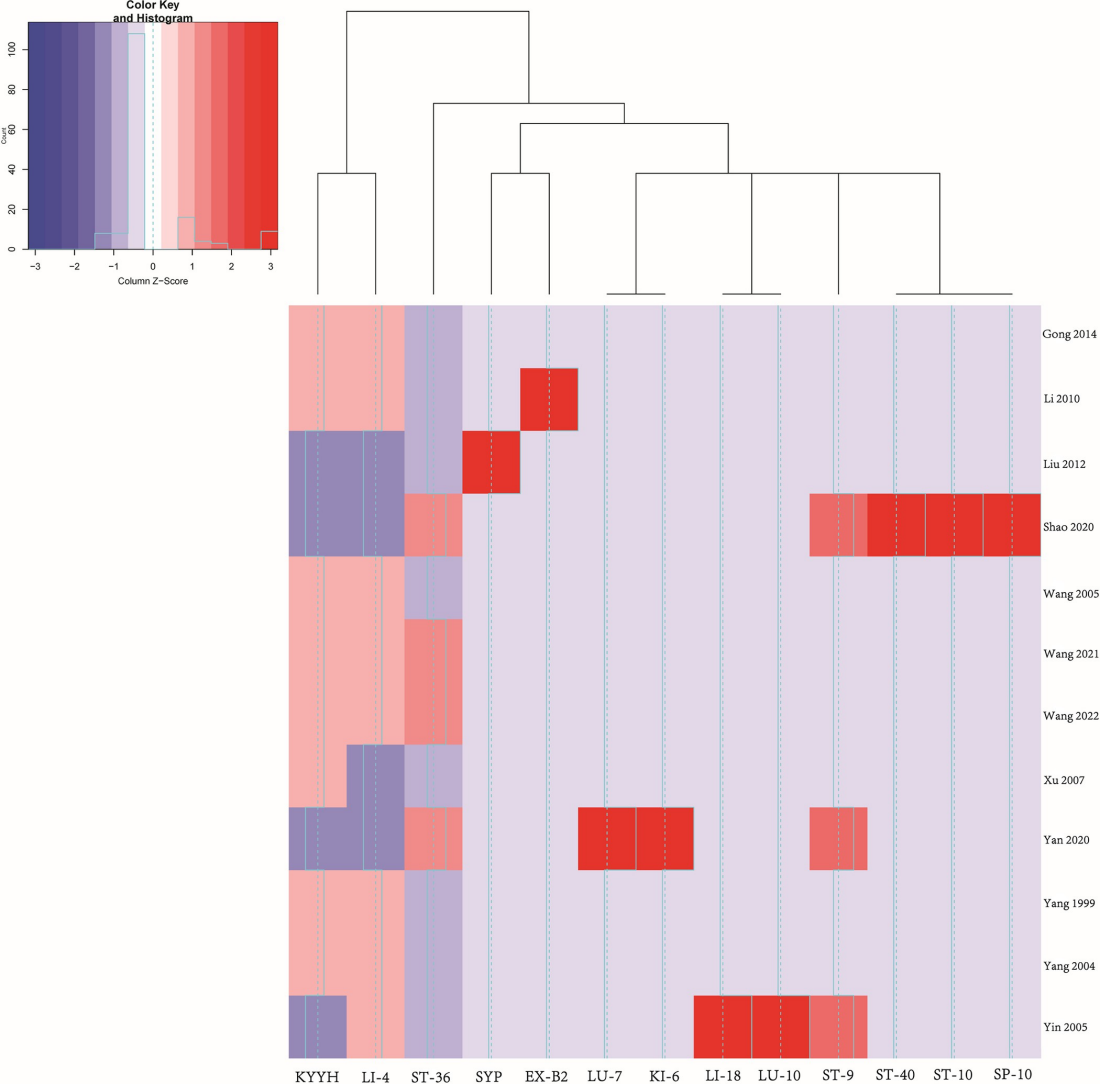
Cluster analyses and heat map of the acupoints applied in the included trials. KYYH: *Kai yin yi hao*; LI-4: He gu; EX-B2: Jia ji; ST-36: Zu san li; LU-7: Lie que; KI-6: Zhao hai; SYP: Sang yin point; LI-18: Fu tu; LU-10: Yu ji; ST-9: Ren yin; ST-40: Feng long; ST-10: Shui tu; SP-10: Xue hai.

As for cluster analyses, results of heat map showed some clustering effects between acupoints selected for acupuncture in the trials. In addition to several one-time application of acupoints [including Shui tu (ST-10), Fu tu (LI-18), Sang yin point, Jia ji (EX-B2), Xue hai (SP-10), Feng long (ST-40), Lie que (LU-7), Zhao hai (KI-6), Yu ji (LU-10)], *Kai yin yi hao* and He gu (LI-4) were the most frequently applied matching-acupoints in trials (as component or alone). Other frequently selected acupoints including Zu san li (ST-36) and Ren yin (ST-9) (Fig 5). Results of correlation coefficient between the acupoints were also showed in S17 Fig.

## Discussion

VN, which is also known as singer’s nodules, teacher’s nodules, or “shouting (caused) nodules” when occurred in children, is a special type of chronic laryngitis induced by inflammatory lesions [3]. The main clinical symptom of VN is hoarseness, and in research with patients were suffering from voice disorders, vocal nodules was diagnosed among 40% of them [31]. However, Shah, et al. revealed that objective and subjective voice measurements were not different in various vocal nodule sizes statistically, other than pitch reduction [32]. Meanwhile, in a study with 79 VN patients showed that there was little evidence to suggest that the nodules themselves were “driving” the severity of the dysphonia [33]. In this review, results of meta-regression based sub-group analyses suggested that sizes of effect were varying in different ranges of ages, genders, duration of disease. As a result, we also believe that the variables above are also associated with sizes of VN, and further researches concerning relationships between different sizes of VN and severities of voice disorder are needed to improve this study and provide more objective evidence of subgroups.

A diagnosis of VN can be made through laryngoscopy clinically, and modern artificial intelligence technologies, such as deep-learning-based computer-aided diagnosis, could provide valuable references for diagnosis of benign, precancerous, and cancer lesions during laryngoscopy examination [34]. Treatments for VN worldwide are divided into two categories, mainly, as surgery and non-surgery therapy, while surgery therapy and drug therapy (such as antibiotic, steroid, or anti-reflux medications) are not routinely recommended in guidelines [35, 36]. Different from considerable one-off expenditure and physical quality requirements of VN surgery, VT has a high status in non-surgical therapies for VN [37]. However, in China and many other developing countries, there are no specific speech pathology specialty, and VT is poorly developed or absent.

Results of this review indicate that compared with oral WM alone and oral CHM alone, pooled results of our study covering 15 trials with 1118 participants favored acupuncture alone concerning higher CER (acupuncture vs. WM, and acupuncture vs. CHM), more reduction on symptom scores (acupuncture vs. CHM), and more improvement on scores of voice analyses (acupuncture vs. CHM). However, VT exhibited favorable improvements compared with acupuncture for VN, including higher CER, and more reduction on scores of GRB and VHI during follow-ups.

Among all of the trials included in this study, acupuncture was performed manually. After pooling all the acupoints applied in the trials, two types of acupoint protocols were noticed, including local acupoints (located at face and neck) and remote acupoints (located at upper and lower limbs). In TCM, primarily, acupoints are classified into 1) acupoints of fourteen meridians, 2) extra acupoints (in addition to the fourteen meridians), and 3) ashi acupoints. For the first and second types, there are names, locations and indications for each of the acupoints, while there is no fixed name, location or indication for ashi acupoints, the third type [38]. Based on these, in terms of general efficacy of certain disorder or disease, the classifications above can be recombined and divided into 1) local (effects) acupoints, 2) remote (effects) acupoints, and 3) *specific* (effects) *acupoints* [39]. Local effects are equipped by all acupoints (including acupoints of fourteen meridians, extra acupoints, and ashi acupoints), such as head acupuncture for post-stroke aphasia, acupuncture at Jingming (BL1) for dry eye disease, and local acupressure for diabetic peripheral neuropathy of lower limbs [40–42]. Remote effects are equipped by acupoints of fourteen meridians and extra acupoints, especially those located at distal end of elbow joints of upper limbs and distal end of knee joints of lower limbs, with effects of their belonging meridians, such as acupuncture at Qu chi (LI-11) and Zu san li (ST-36) for left hemiplegia after ischemic stroke, and wrist-ankle acupuncture for hypertension after intubation during induction of general anesthesia [43, 44]. In addition, from clinical experience to medical evidence, in thousands of years, many *specific acupoints* were discovered, tested, and summarized for acupuncture with good efficacy, long duration, fewer needles, such as acupuncturing at sphenopalatine ganglion acupoint for allergic rhinitis [45]. In this review, cluster analyses and correlation coefficient of the acupoints revealed that *Kai yin yi hao* and He gu (LI-4) were the most frequently applied matching-acupoints in the included trials, followed by Zu san li (ST-36) and Ren yin (ST-9). Commonly, local acupoints are selected and matched with remote and (or) *specific acupoints* in acupuncture, which is beneficial for achieving enough acupuncture stimulation, longer efficacy duration, and balanced meridian effects per treatment [46]. Our results of meta-regression for CER (acupuncture vs. WM and acupuncture vs. CHM) also suggested superiorities of local acupoints plus remote acupoints, compared with local acupoints applied alone.

In our study, only six of the included trials reported that there was no acupuncture specific adverse event, while it was not mentioned in other nine trials. Sometimes, patients may feel local and mild sensations of sour, numb, distension, painful during or (and) after acupuncture. The sensations are associated with *De Qi* (obtaining *Qi*) in TCM, and will clear up several days later without specific medical care required [47]. Such mild and self-limited events include local bruising and radiating pain. In addition, severer events may appear on very few first-time acupuncture patients due to fasting state, uncomfortable position, overstrain, or movement during acupuncture, including fainting, sticking of needle, broken of needle, vascular injury, nervous system injury, or even visceral injury [48]. As a result, detailed communication between doctors or acupuncture practitioners and patients before acupuncture, required preparations, and complete post treatment orders are necessary.

As for study quality and risk of bias, all the 15 trials are RCTs, but none of them implied placebo control. Randomization method was clear and appropriate in 10 trials, while it was in unclear risk of bias for the other 5 trials. Allocation concealment and blinding (for outcome assessment) method were of unclear risk of bias in all trials. No study reported drop-out, and a protocol or registration ahead of experiment was not mentioned. Publication bias (by Egger’s tests) or instability (by trim and fill tests) was not suspected in results, and more inclusions were required to meet the required information size for all comparisons according to trial sequential analyses and penalized tests. According to the GRADE, levels of evidence were moderate (⨁⨁⨁◯, GRADE B) concerning acupuncture vs. CHM, but were low (⨁⨁◯◯, GRADE C) concerning acupuncture vs. WM and acupuncture vs. VT. As a result, more placebo or blank-controlled, double-blind, prospective, randomized trials of acupuncture for VN are urgently needed.

## Conclusion

Our study, the first one with RCT evidence from 15 trials involving 1,118 participants, proved that applying acupuncture yielded better improvement for patients with VN compared with WM and CHM. However, negative results were discovered in acupuncture vs. VT groups in this study. There is also need for RCTs with improvements on designing and interventions in experimental and controls.

### Limitations

This study had several limitations. Firstly, most of the trials included were of moderate to high risk of bias, with reasons such as without mentioning details of random sequence generation method, allocation concealment, and blinding of participants, personnel and outcome assessment. This is the main reason for low quality of the included trials. Secondly, interventions and follow-up periods were short among most of the trials, while longer treatment duration and follow-up periods for VN, a chronic and recurrent disorder, is essential and required. Finally, inclusion of trials and participants were limited due to the few numbers of published trials and small sample sizes, relatively.

## Supporting information

S1 Checklist PRISMA 2020 checklist. (DOCX)

S1 Fig Risk of bias graph. (PDF)

S2 Fig Risk of bias summary. (PDF)

S3 Fig Meta-influence analysis for acupuncture vs. western medicine on clinical effective rate. (PDF)

S4 Fig Meta-regression for acupuncture vs. western medicine on clinical effective rate. (PDF)

S5 Fig Forest plot for acupuncture vs. Chinese herbal medicine on reduction symptom scores. (PDF)

S6 Fig Forest plot for acupuncture vs. Chinese herbal medicine on improvement on scores of voice analyses. (PDF)

S7 Fig Forest plot for acupuncture vs. voice training on clinical effective rate. (PDF)

S8 Fig Forest plot for acupuncture vs. voice training on reduction on scores of grade, roughness, and breathiness (one month after treatment). (PDF)

S9 Fig Forest plot for acupuncture vs. voice training on reduction on scores of grade, roughness, and breathiness (two months after treatment). (PDF)

S10 Fig Forest plot for acupuncture vs. voice training on reduction on voice handicap index (one month after treatment). (PDF)

S11 Fig Forest plot for acupuncture vs. voice training on reduction on voice handicap index (two months after treatment). (PDF)

S12 Fig Trial sequential analysis and penalized test for acupuncture vs. Chinese herbal medicine on clinical effective rate. (PNG)

S13 Fig Egger’s test for acupuncture vs. western medicine on clinical effective rate. (PDF)

S14 Fig Egger’s test for acupuncture vs. Chinese herbal medicine on clinical effective rate. (PDF)

S15 Fig Trim and fill test for acupuncture vs. western medicine on clinical effective rate. (PDF)

S16 Fig Trim and fill test for acupuncture vs. Chinese herbal medicine on clinical effective rate. (PDF)

S17 Fig Analyses on correlation coefficient of the acupoints applied in the included trials. KYYH: *Kai yin yi hao*; LI-4: He gu; EX-B2: Jia ji; ST-36: Zu san li; LU-7: Lie que; KI-6: Zhao hai; SYP: Sang yin point; LI-18: Fu tu; LU-10: Yu ji; ST-9: Ren yin; ST-40: Feng long; ST-10: Shui tu; SP-10: Xue hai.
(TIFF)

S1 Table Detailed characteristics of the included trials. E: Experimental; C: Control; M: Male; F: Female; Y: Year; M: Month; W: Week; D: Day; NR: Not reported; WM: Western medicine; VT: Voice training; CHM: Chinese herbal medicine.
(DOCX)

S2 Table Summary of acupoints applied and frequency in the included trials. *A pair of experience acupoints located at one *cun* beside prominentia laryngea in the neck (one *cun* outward from the notch of thyroid cartilage), and close to the lateral edge of thyroid cartilage;**A pair of experience acupoints located at center of the thyrohyoid membrane. One *cun* up from Ren yin (ST-9) and one *cun* to both sides;***From the 3^rd^ cervical vertebra to the 5^th^ cervical vertebra.
(DOCX)

S3 Table Summary of meta-regression and subgroup-analyses for clinical effective rate (acupuncture vs. Chinese herbal medicine). RR: Risk Ratio; qd: Once per day; Ref: Reference. *The values were mean or median.
(DOCX)

S4 Table Strength of evidence recommendations by the grading of recommendations assessment, development and evaluation system. CI: Confidence interval; RR: Risk ratio; WMD: Weighted mean difference; GRB: A tool for evaluation of hoarseness, including Grade (overall grade of hoarseness), Roughness (roughness of voice), and Breathiness (breathiness of voice) by doctors; VHI: Voice handicap indexo.
(DOCX)

S1 File Search terms applied in review. (PDF)

## Abbreviations

VN: Vocal nodules
VT: Voice training
CHM: Chinese herbal medicine
TCM: Traditional Chinese medicine
WM: Western medicine
CER: Clinical effective rate
GRB: Grade, roughness, breathiness
VHI: Voice handicap index
RR: Risk ratio
WMD: Weighted mean difference
CI: Confidence interval
GRADE: The Grading of Recommendations Assessment, Development and Evaluation system
