# Supplementary figures and images for: Efficacy and safety of acupuncture for vocal nodules: A systematic review and meta-analysis with trial sequential analysis
Source: PLoS One. 2023 Nov 3;18(11):e0288252. doi: 10.1371/journal.pone.0288252 (PMC10624316; doi:10.1371/journal.pone.0288252)

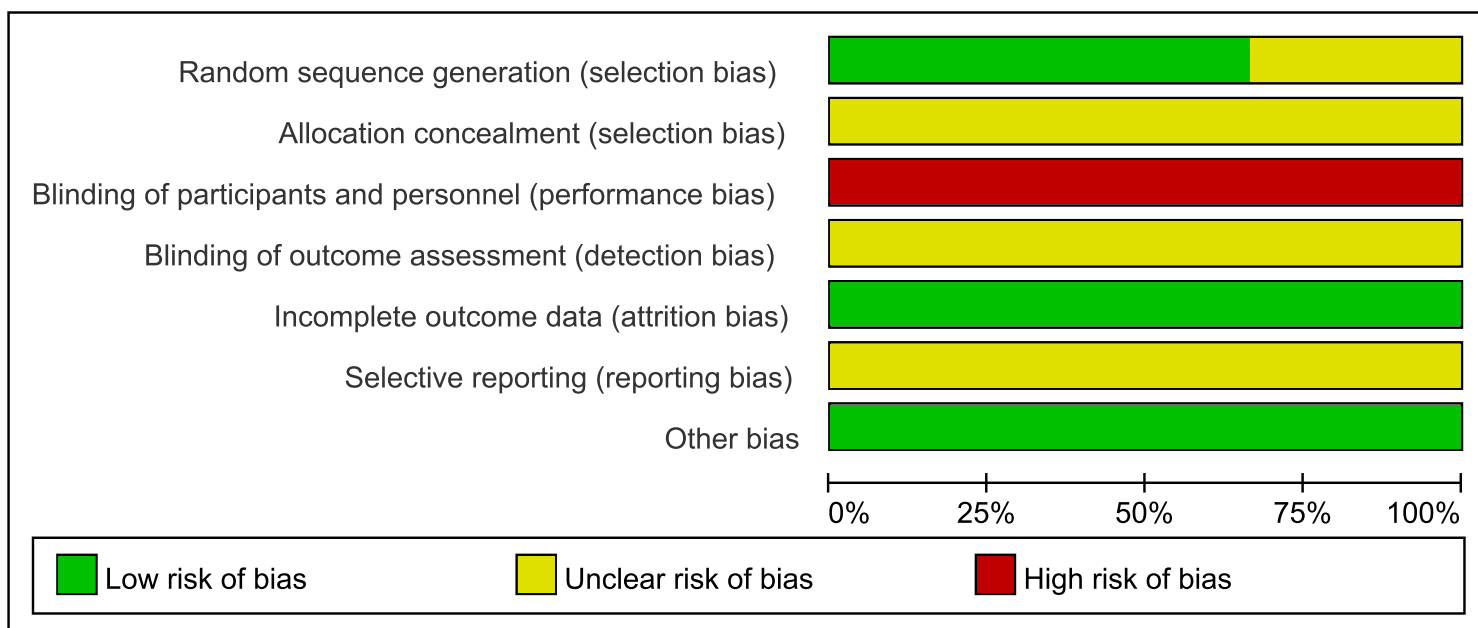

Supplement: S1 Fig — (PDF) [file pone.0288252.s002.pdf]

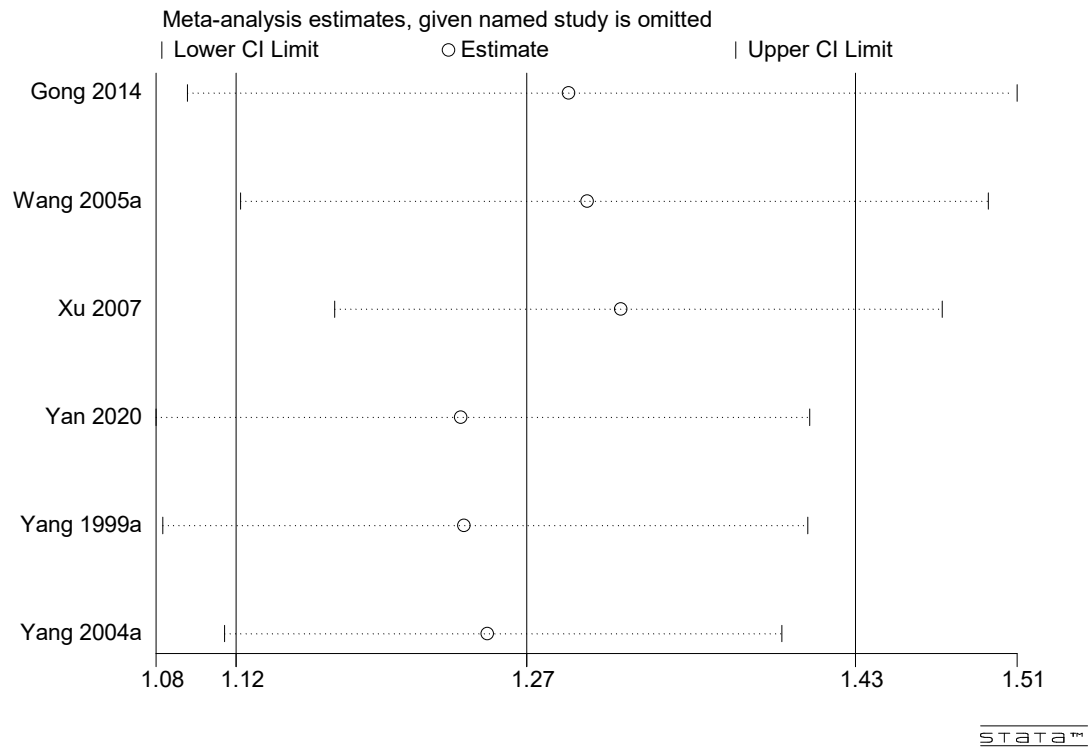

Supplement: S3 Fig — (PDF) [file pone.0288252.s004.pdf]

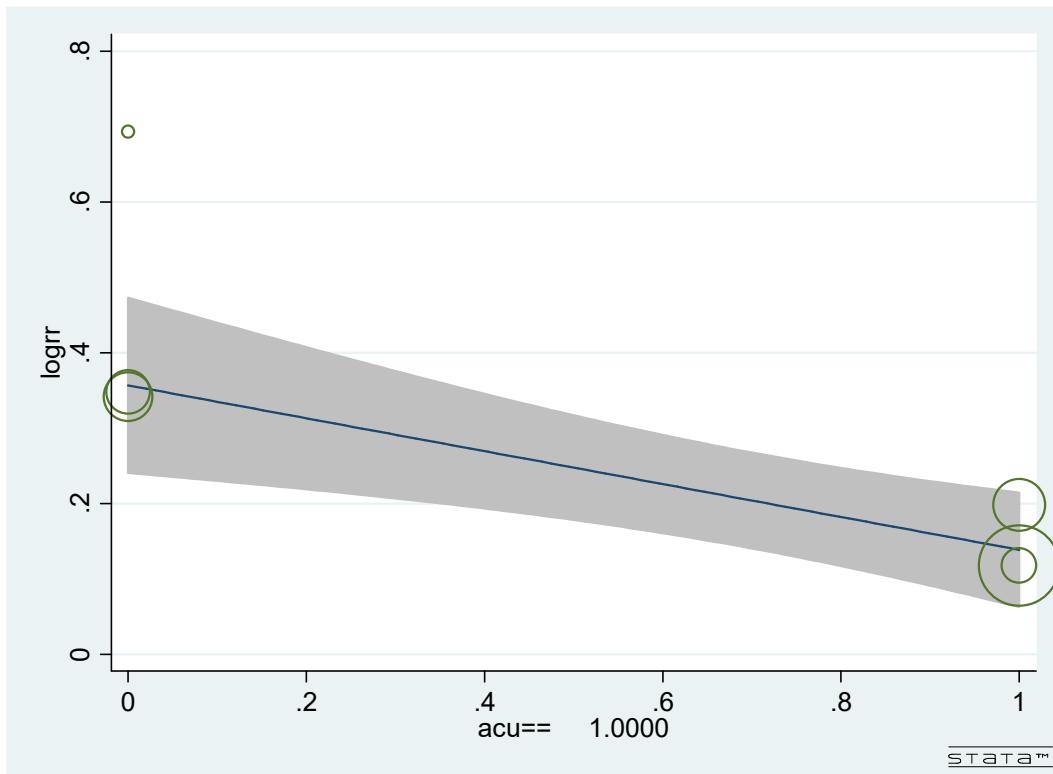

Supplement: S4 Fig — (PDF) [file pone.0288252.s005.pdf]

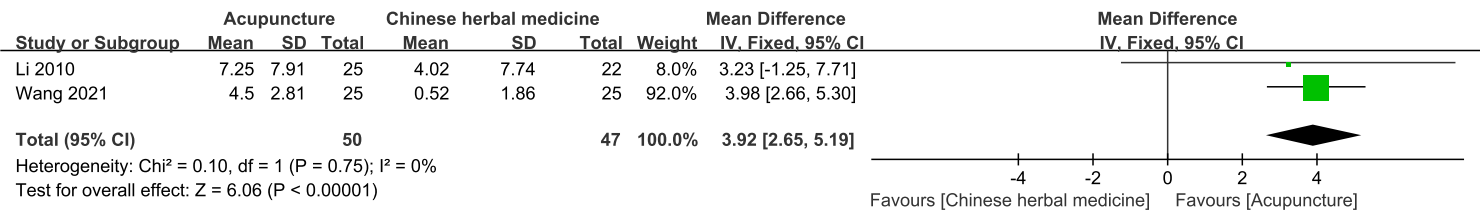

Supplement: S5 Fig — (PDF) [file pone.0288252.s006.pdf]

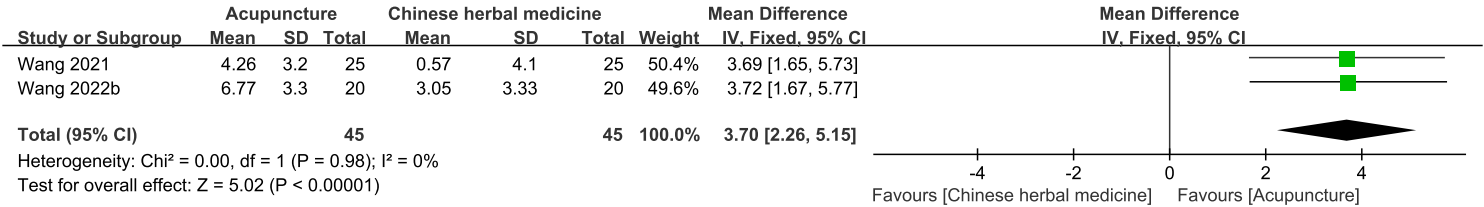

Supplement: S6 Fig — (PDF) [file pone.0288252.s007.pdf]

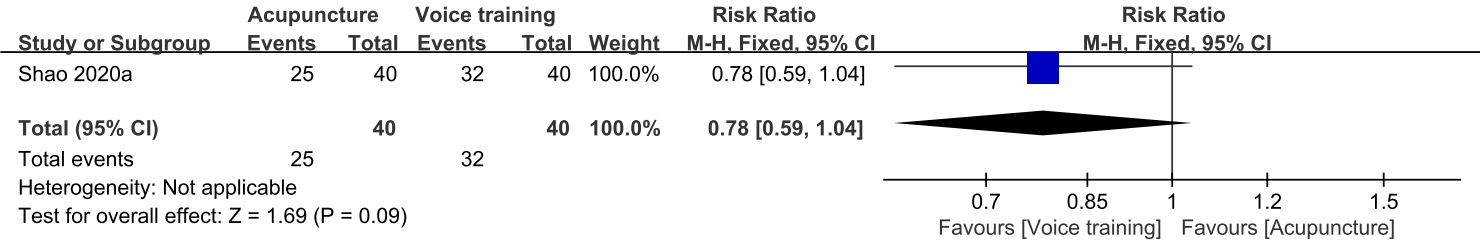

Supplement: S7 Fig — (PDF) [file pone.0288252.s008.pdf]

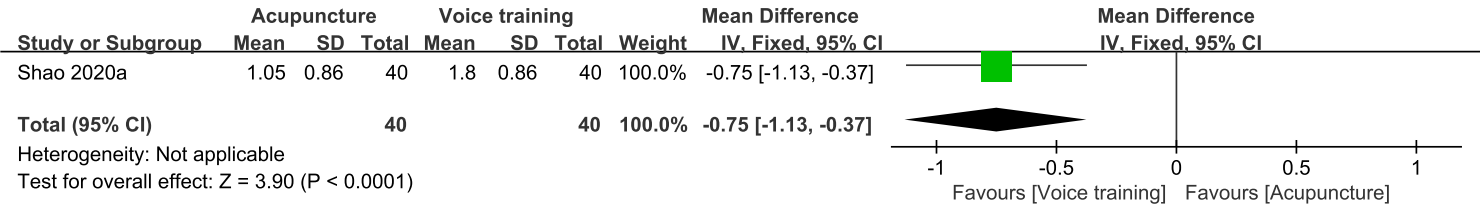

Supplement: S8 Fig — (PDF) [file pone.0288252.s009.pdf]

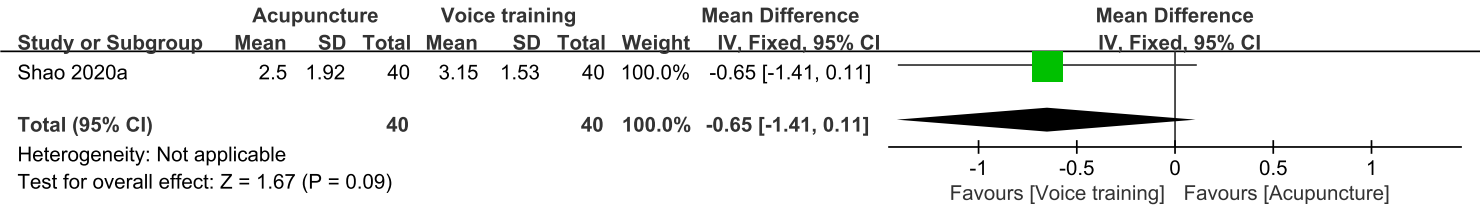

Supplement: S9 Fig — (PDF) [file pone.0288252.s010.pdf]

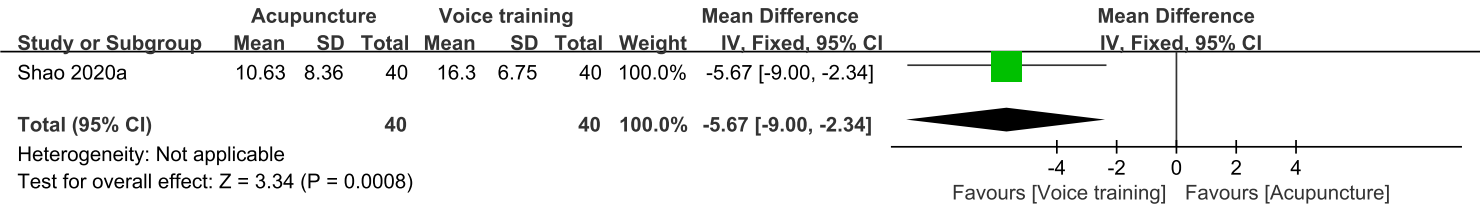

Supplement: S10 Fig — (PDF) [file pone.0288252.s011.pdf]

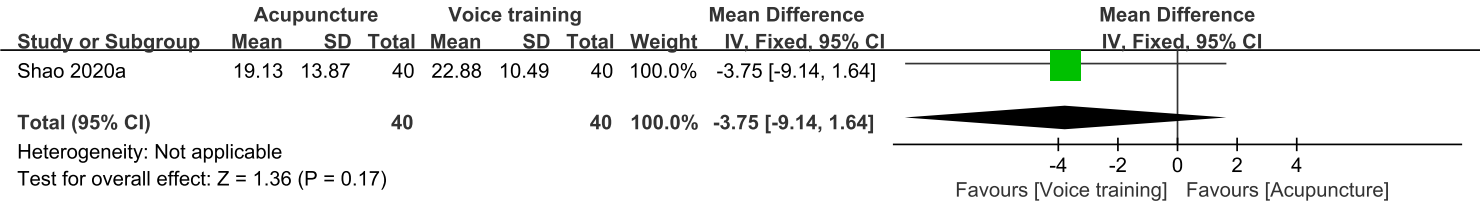

Supplement: S11 Fig — (PDF) [file pone.0288252.s012.pdf]

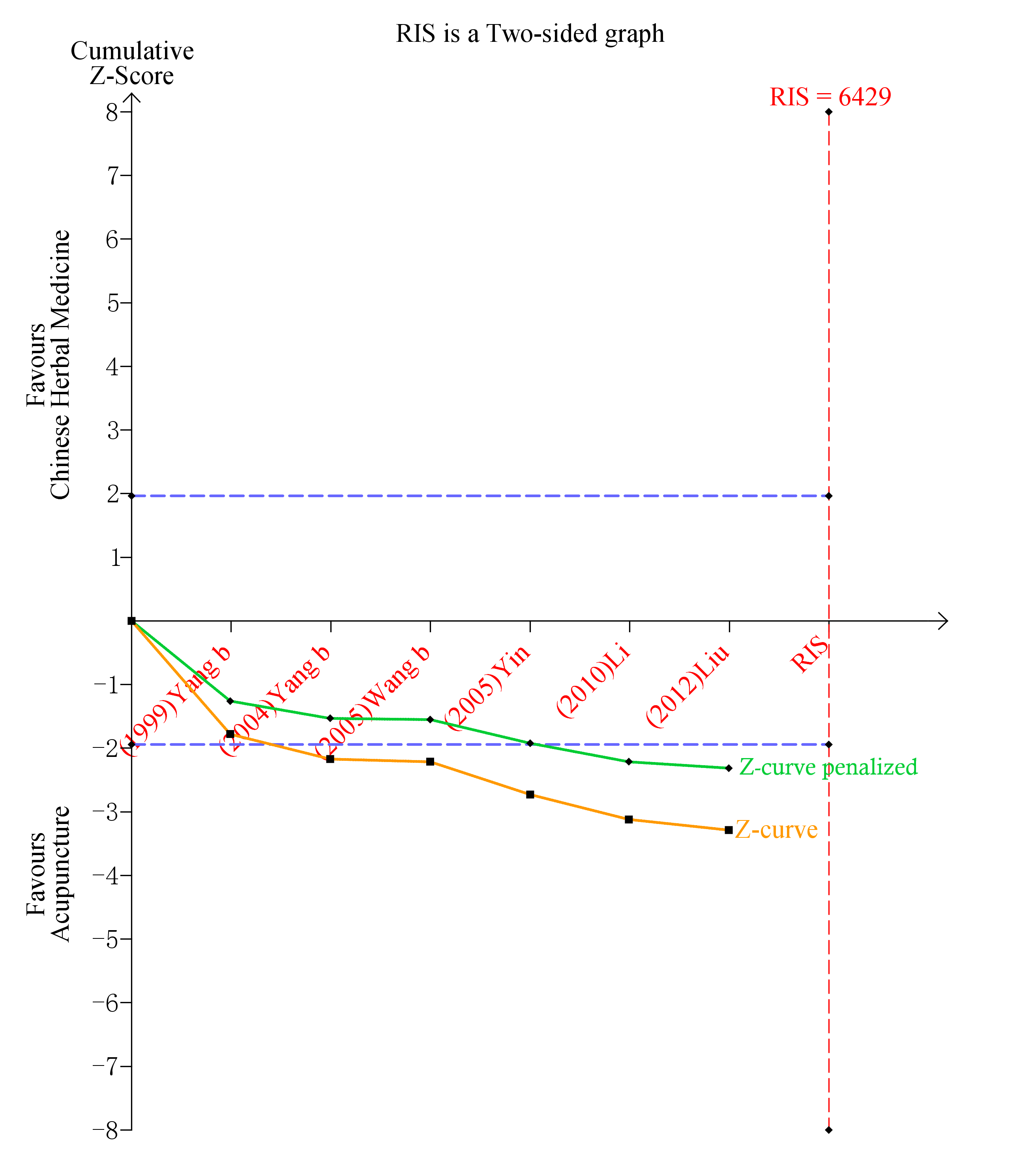

Supplement: S12 Fig — (PNG) [file pone.0288252.s013.png]

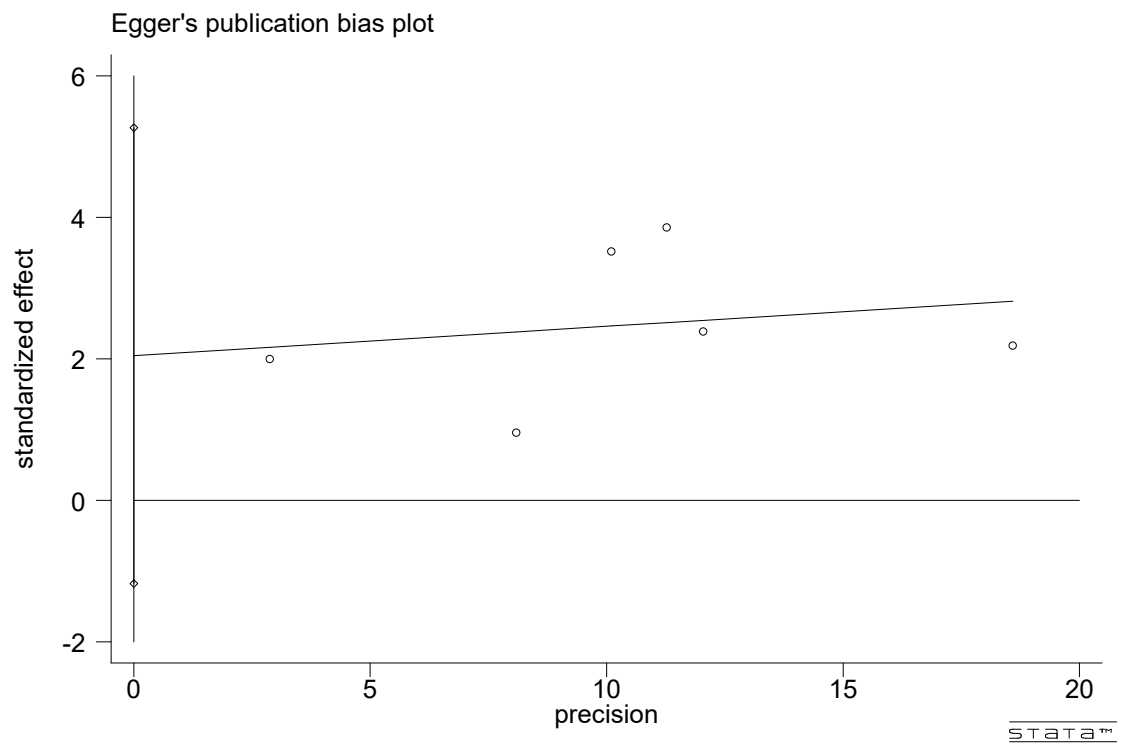

Supplement: S13 Fig — (PDF) [file pone.0288252.s014.PDF]

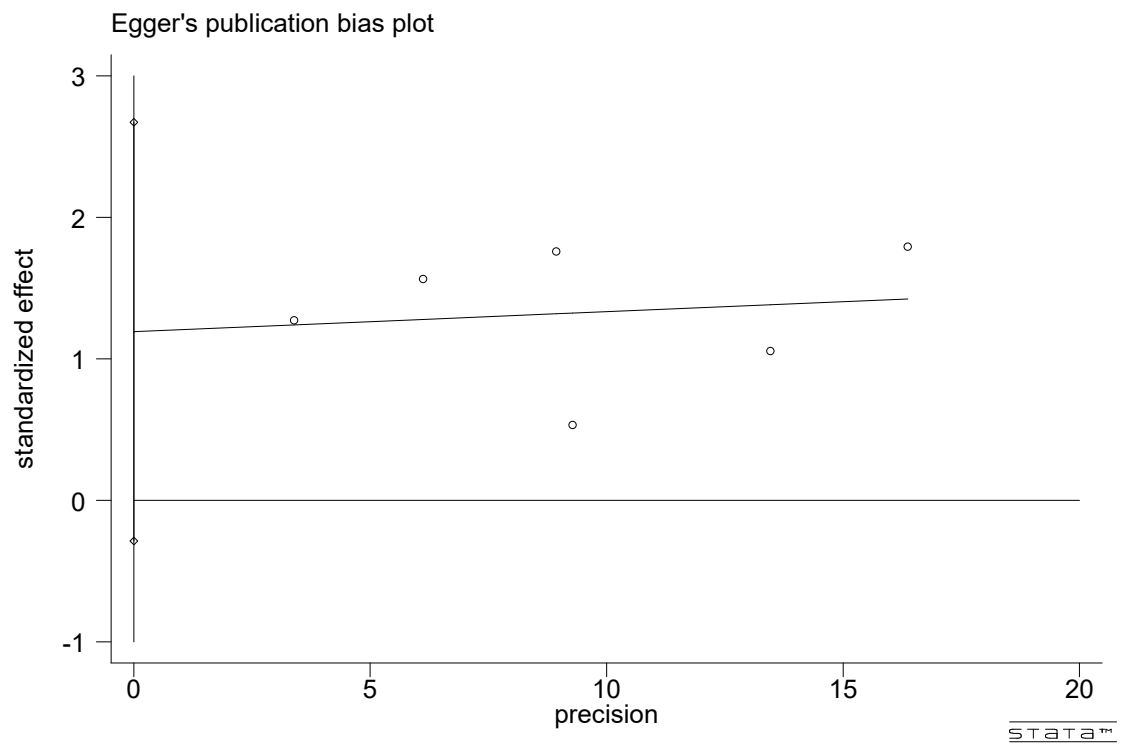

Supplement: S14 Fig — (PDF) [file pone.0288252.s015.pdf]

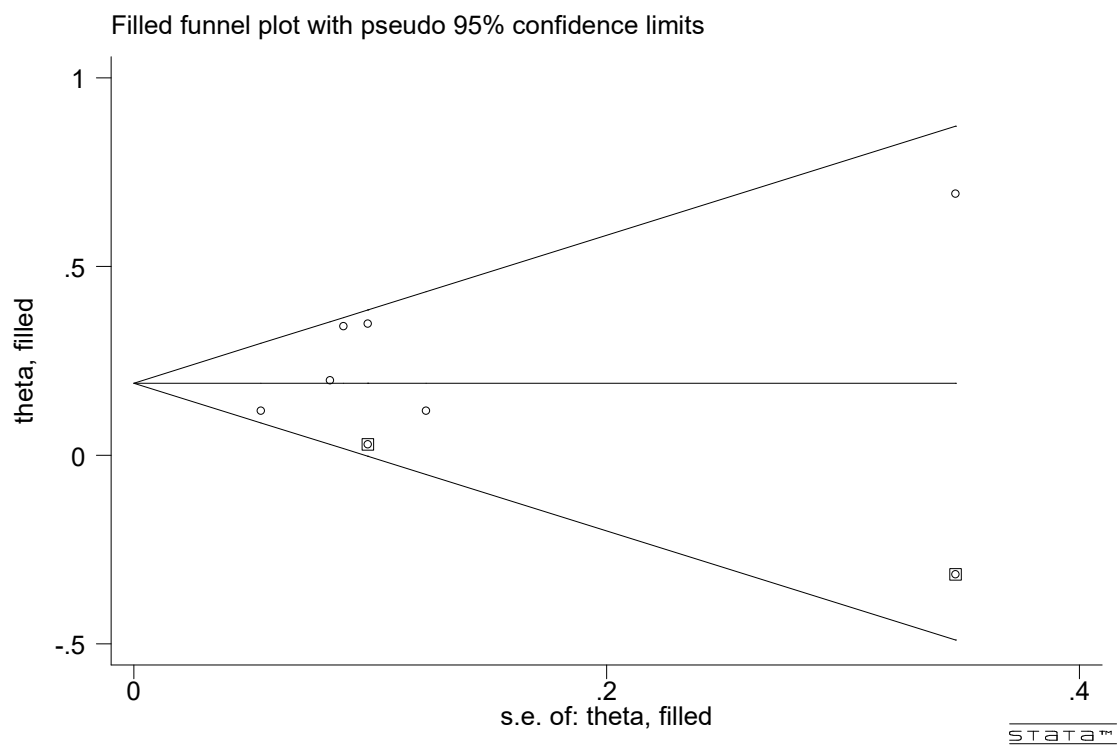

Supplement: S15 Fig — (PDF) [file pone.0288252.s016.pdf]

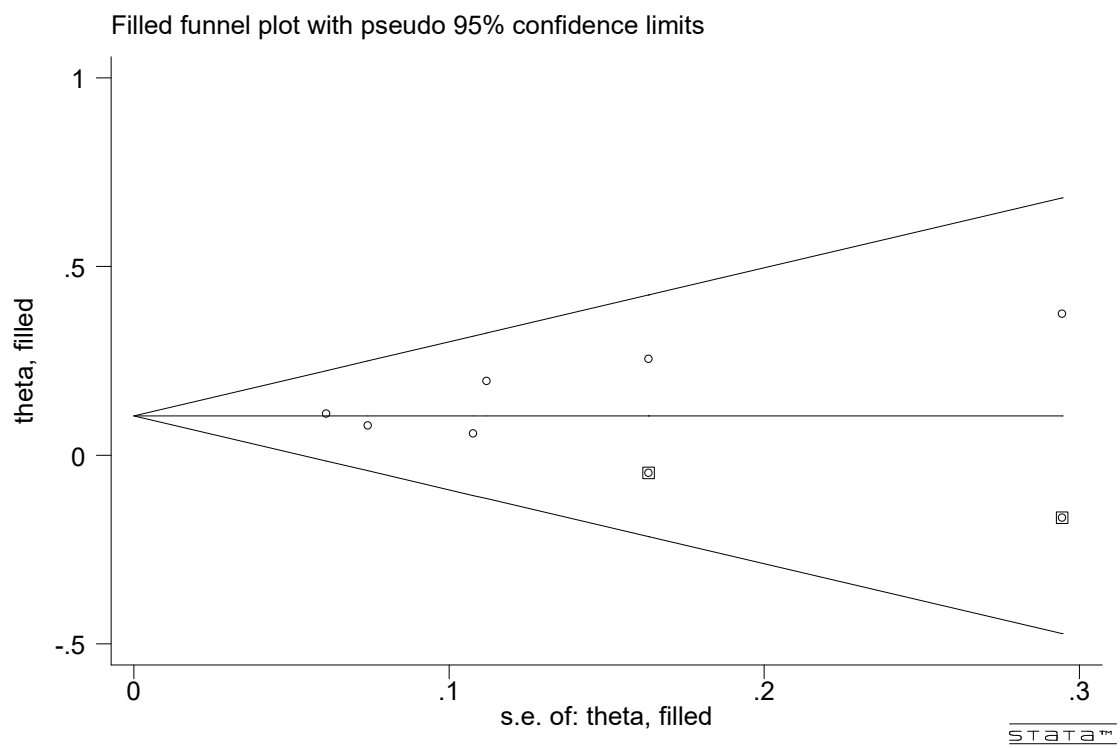

Supplement: S16 Fig — (PDF) [file pone.0288252.s017.pdf]
