# Supplementary material for: Efficacy and safety of acupuncture for vocal nodules: A systematic review and meta-analysis with trial sequential analysis
Source: PLoS One. 2023 Nov 3;18(11):e0288252. doi: 10.1371/journal.pone.0288252 (PMC10624316; doi:10.1371/journal.pone.0288252)
Supplement: S2 Fig — (PDF) [file pone.0288252.s003.pdf]

	Random sequence generation (selection bias)	Allocation concealment (selection bias)	Blinding of participants and personnel (performance bias)	Blinding of outcome assessment (detection bias)	Incomplete outcome data (attrition bias)	Selective reporting (reporting bias)	Other bias
Gong 2014	?	?	−	?	+	?	+
Li 2010	+	?	−	?	+	?	+
Liu 2012	+	?	−	?	+	?	+
Shao 2020a	+	?	−	?	+	?	+
Wang 2005a	+	?	−	?	+	?	+
Wang 2005b	+	?	−	?	+	?	+
Wang 2021	+	?	−	?	+	?	+
Wang 2022b	+	?	−	?	+	?	+
Xu 2007	?	?	−	?	+	?	+
Yan 2020	?	?	−	?	+	?	+
Yang 1999a	?	?	−	?	+	?	+
Yang 1999b	?	?	−	?	+	?	+
Yang 2004a	+	?	−	?	+	?	+
Yang 2004b	+	?	−	?	+	?	+
Yin 2005	+	?	−	?	+	?	+
