# Supplementary material for: Efficacy and safety of acupuncture for vocal nodules: A systematic review and meta-analysis with trial sequential analysis
Source: PLoS One. 2023 Nov 3;18(11):e0288252. doi: 10.1371/journal.pone.0288252 (PMC10624316; doi:10.1371/journal.pone.0288252)
Supplement: S1 Table — E: Experimental; C: Control; M: Male; F: Female; Y: Year; M: Month; W: Week; D: Day; NR: Not reported; WM: Western medicine; VT: Voice training; CHM: Chinese herbal medicine. (DOCX) [file pone.0288252.s019.docx]

| Study | Sample-E (M/F), cases | Sample-C (M/F), cases | Age (E/C), years | Course of disease (E/C) | Basis of diagnosis | Intervention-C | Frequency of treatment (E/C) | Course of treatment | Follow-ups | Deqi | Acupoint selection |
| --- | --- | --- | --- | --- | --- | --- | --- | --- | --- | --- | --- |
| Yan 2020 | 42/42 | 40/44 | 40.1±0.4/38.5 ± 0.4 | 0.9±0.5/0.8±0.5,Y | Laryngoscope | WM | 15 times in 20 D/bid | 20D | 2M | Yes | Local and remote acupoints |
| Shao 2020 b | 26/14 | 30/10 | 36.7±9.519/38.85±8.481 | 6.55±2.132/7.1±1.546,W | Laryngoscope | VT | qd/qw | 2M | NR | Yes | Local and remote acupoints |
| Wang 2005 a | 16/24 | 9/11 | 12-65(36)/12-63(33) | 4M-2Y/3M-2Y | NR | WM | tid/bid | 1M | NR | Yes | Local acupoints only |
| Wang 2005 b | 16/24 | 7/13 | 12-65(36)/11-68(35) | 4M-2Y/3M-2Y | NR | CHM | tid/bid | 1M | NR | Yes | Local acupoints only |
| Wang 2021 | 6/19 | 8/17 | 32.1±9.5/31.9±10.2 | 7.3 ± 3.5M/7.6 ± 2.7M | Stroboscopic laryngoscope | CHM | biw/bid | 2M | 6M | NR | Local and remote acupoints |
| Wang 2022 b | 11/9 | 8/12 | 38.40 ± 4.73/39.58±3.78 | 1.19±0.32/1.27±0.44,Y | Laryngoscope | CHM | biw/bid | 1M | 6M | Yes | Local and remote acupoints |
| Xu 2007 | 49/30 | 35/25 | 30.6 | 2M-5Y | Laryngoscope | WM | qd/qd | 18D | NR | NR | Local acupoints only |
| Li 2010 | 10/15 | 8/14 | 34.17±12.54/36.89±11.07 | 6.78±4.21/7.54±5.13,M | Laryngoscope | CHM | qod/tid | 20D | NR | Yes | Local and remote acupoints |
| Gong 2014 | 19/21 | 18/22 | 36±2/33±2 | NR | Laryngoscope | WM | qd/qd | 1M | NR | Yes | Local acupoints only |
| Yang 2004 a | 10/20 | 12/18 | 18-65/18-60 | 1-3,M: 21 cases; 4-6,M: 9 cases/1-3,M: 17 cases; 4-6,M: 13 cases | Indirect laryngoscope | WM | qd/qd | 1M | NR | NR | Local and remote acupoints |
| Yang 2004 b | 10/20 | 9/21 | 18-65/19-61 | 1-3,M: 21 cases; 4-6,M: 9 cases/1-3,M: 19 cases; 4-6,M: 11 cases | Indirect laryngoscope | CHM | qd/tid | 1M | NR | NR | Local and remote acupoints |
| Liu 2012 | 11/18 | 10/18 | 20-57 | >3M | Laryngoscope | CHM | qd/tid | 1M | NR | Yes | Local acupoints only |
| Yin 2005 | 5/25 | 6/24 | 18-48/18-46 | 25D-1Y/20D-1Y | Indirect laryngoscope | CHM | qd/bid | 21D | NR | Yes | Local and remote acupoints |
| Yang 1999 a | 51/58 | 27/29 | 17-46(27.5)/16-44(28.7) | 1-6/1-5,Y | NR | WM | qod/qd | 20D | NR | NR | Local and remote acupoints |
| Yang 1999 b | 51/58 | 30/32 | 17-46(27.5)/17-45(29.2) | 1-6/1-6,Y | NR | CHM | qod/6 per D | 20D | NR | NR | Local and remote acupoints |

E: Experimental; C: Control; M: Male; F: Female; Y: Year; M: Month; W: Week; D: Day; NR: Not reported; WM: Western medicine; VT: Voice training; CHM: Chinese herbal medicine.
