# Supplementary material for: Efficacy and safety of acupuncture for vocal nodules: A systematic review and meta-analysis with trial sequential analysis
Source: PLoS One. 2023 Nov 3;18(11):e0288252. doi: 10.1371/journal.pone.0288252 (PMC10624316; doi:10.1371/journal.pone.0288252)
Supplement: S2 Table — *A pair of experience acupoints located at one cun beside prominentia laryngea in the neck (one cun outward from the notch of thyroid cartilage), and close to the lateral edge of thyroid cartilage;**A pair of experience acupoints located at center of the thyrohyoid membrane. One cun up from Ren yin (ST-9) and one cun to both sides;***From the 3rd cervical vertebra to the 5th cervical vertebra. (DOCX) [file pone.0288252.s020.docx]

| Classification | Acupoint | Frequency |
| --- | --- | --- |
| Local acupoints | Kai yin yi hao (Exp)* | 8 |
|  | Ren yin (ST-9) | 3 |
|  | Shui tu (ST-10) | 1 |
|  | Fu tu (LI-18) | 1 |
|  | Sang yin point (Exp)** | 1 |
|  | Jia ji (EX-B2)*** | 1 |
| Remote acupoints | He gu (LI-4) | 8 |
|  | Zu san li (ST-36) | 4 |
|  | Xue hai (SP-10) | 1 |
|  | Feng long (ST-40) | 1 |
|  | Lie que (LU-7) | 1 |
|  | Zhao hai (KI-6) | 1 |
|  | Yu ji (LU-10) | 1 |

*A pair of experience acupoints located at one *cun* beside prominentia laryngea in the neck (one *cun* outward from the notch of thyroid cartilage), and close to the lateral edge of thyroid cartilage;**A pair of experience acupoints located at center of the thyrohyoid membrane. One *cun* up from Ren yin (ST-9) and one *cun* to both sides;***From the 3^rd^ cervical vertebra to the 5^th^ cervical vertebra.
