# Supplementary material for: Efficacy and safety of acupuncture for vocal nodules: A systematic review and meta-analysis with trial sequential analysis
Source: PLoS One. 2023 Nov 3;18(11):e0288252. doi: 10.1371/journal.pone.0288252 (PMC10624316; doi:10.1371/journal.pone.0288252)
Supplement: S3 Table — RR: Risk Ratio; qd: Once per day; Ref: Reference. *The values were mean or median. (DOCX) [file pone.0288252.s021.docx]

| Characteristics | Meta-regression | | | | Subgroup analysis | | | |
| --- | --- | --- | --- | --- | --- | --- | --- | --- |
|  | tau^2^ | I^2^_resid_ | Adjusted R^2^ | P value | No. of trials | RR (95% CI) | P value of effect size | I^2^ |
| Age, years* | | | | | | | | |
| 30±2.5 | 0 | 0% | 0% | 0.73 | 2 | 1.14 (1.03, 1.27) | 0.014 | 0% |
| 35±2.5 |  |  |  | 0.87 | 2 | 1.16 (0.96, 1.39) | 0.124 | 10.1% |
| 40±2.5 | Ref | | | | 2 | 1.19 (0.96, 1.48) | 0.106 | 55.1% |
| Gender (proportion of male/female), rank | | | | | | | | |
| The top 50% of trials (higher proportions) | 0 | 0% | 0% | 0.87 | 3 | 1.20 (1.03, 1.41) | 0.021 | 19.6% |
| The final 50% of trials (lower proportions) |  |  |  |  | 3 | 1.13 (1.02, 1.25) | 0.018 | 0% |
| Duration of disease, year* | | | | | | | | |
| < 1 | 0 | 0% | 0% | 0.29 | 3 | 1.29 (1.06, 1.57) | 0.01 | 0% |
| ≥ 1 |  |  |  |  | 2 | 1.10 (0.99, 1.22) | 0.07 | 0% |
| Frequency of treatment (acupuncture) * | | | | | | | | |
| qd | 0 | 0% | 0% | 0.87 | 3 | 1.20 (1.03, 1.41) | 0.021 | 19.6% |
| Fewer than qd |  |  |  |  | 3 | 1.13 (1.02, 1.25) | 0.018 | 0% |
| Course of treatment (acupuncture), month* | | | | | | | | |
| <1 | 0 | 0% | 0% | 0.50 | 3 | 1.16 (1.05, 1.29) | 0.004 | 0% |
| 1 |  |  |  |  | 3 | 1.14 (0.98, 1.34) | 0.096 | 0% |
| Selection of acupoints | | | | | | | | |
| Local acupoints only | 0 | 0% | 0% | 0.39 | 3 | 1.07 (0.94, 1.21) | 0.286 | 0% |
| Local and remote acupoints |  |  |  |  | 3 | 1.19 (1.07, 1.33) | 0.002 | 0% |
| Report of *Deqi* (obtaining qi in traditional Chinese medicine) during acupuncture | | | | | | | | |
| Yes | 0 | 0% | 0% | 0.95 | 3 | 1.15 (1.03, 1.28) | 0.012 | 0% |
| Not reported |  |  |  |  | 3 | 1.16 (1.02, 1.33) | 0.029 | 5% |

RR: Risk Ratio; qd: Once per day; Ref: Reference. *The values were mean or median
