# Supplementary material for: Efficacy and safety of acupuncture for vocal nodules: A systematic review and meta-analysis with trial sequential analysis
Source: PLoS One. 2023 Nov 3;18(11):e0288252. doi: 10.1371/journal.pone.0288252 (PMC10624316; doi:10.1371/journal.pone.0288252)
Supplement: S4 Table — CI: Confidence interval; RR: Risk ratio; WMD: Weighted mean difference; GRB: A tool for evaluation of hoarseness, including Grade (overall grade of hoarseness), Roughness (roughness of voice), and Breathiness (breathiness of voice) by doctors; VHI: Voice handicap indexo. (DOCX) [file pone.0288252.s022.docx]

| Certainty assessment | | | | | | | Summary of findings | | | | |
| --- | --- | --- | --- | --- | --- | --- | --- | --- | --- | --- | --- |
| Participants (studies) Follow-up | Risk of bias | Inconsistency | Indirectness | Imprecision | Publication bias | Overall certainty of evidence | Study event rates (%) | | Relative effect (95% CI) | Anticipated absolute effects | |
|  |  |  |  |  |  |  | With placebo | With Clinical Effective Rate |  | Risk with placebo | Risk difference with Clinical Effective Rate |
| Clinical effective rate (Acupuncture vs. Western medicine) | | | | | | | | | | | |
| 672 (6 RCTs) | serious | serious | not serious | not serious | none | ⨁⨁◯◯ Low | 199/290 (68.6%) | 346/382 (90.6%) | RR 1.27 (1.12 to 1.43) | 686 per 1,000 | 185 more per 1,000 (from 82 more to 295 more) |
| Clinical effective rate (Acupuncture vs. Chinese herbal medicine) | | | | | | | | | | | |
| 455 (6 RCTs) | serious | not serious | not serious | not serious | none | ⨁⨁⨁◯ Moderate | 143/192 (74.5%) | 232/263 (88.2%) | RR 1.16 (1.06 to 1.26) | 745 per 1,000 | 119 more per 1,000 (from 45 more to 194 more) |
| Reduction on symptom scores (Acupuncture vs. Chinese herbal medicine) | | | | | | | | | | | |
| 97 (2 RCTs) | serious | not serious | not serious | not serious | none | ⨁⨁⨁◯ Moderate | 47 | 50 | - | The mean reduction on symptom scores (Acupuncture vs. Chinese herbal medicine) was 0 | WMD 3.92 higher (2.65 higher to 5.19 higher) |
| Improvement on scores of voice analyses (Acupuncture vs. Chinese herbal medicine) | | | | | | | | | | | |
| 90 (2 RCTs) | serious | not serious | not serious | not serious | none | ⨁⨁⨁◯ Moderate | 45 | 45 | - | The mean improvement on scores of voice analyses (Acupuncture vs. Chinese herbal medicine) was 0 | WMD 3.7 higher (2.26 higher to 5.15 higher) |
| Clinical effective rate (Acupuncture vs. Voice training) | | | | | | | | | | | |
| 80 (1 RCT) | serious | not serious | not serious | serious | none | ⨁⨁◯◯ Low | 32/40 (80.0%) | 25/40 (62.5%) | RR 0.78 (0.59 to 1.04) | 800 per 1,000 | 176 fewer per 1,000 (from 328 fewer to 32 more) |
| Reduction on scores of GRB (Acupuncture vs. Voice Training, one month after treatment) | | | | | | | | | | | |
| 80 (1 RCT) | serious | not serious | not serious | serious | none | ⨁⨁◯◯ Low | 40 | 40 | - | The mean reduction on scores of GRB (Acupuncture vs. Voice Training, one month after treatment) was 0 | WMD 0.75 lower (1.13 lower to 0.37 lower) |
| Reduction on scores of GRB (Acupuncture vs. Voice Training, two months after treatment) | | | | | | | | | | | |
| 80 (1 RCT) | serious | not serious | not serious | serious | none | ⨁⨁◯◯ Low | 40 | 40 | - | The mean reduction on scores of GRB (Acupuncture vs. Voice Training, two months after treatment) was 0 | WMD 0.65 lower (1.41 lower to 0.11 higher) |
| Reduction on scores of VHI (Acupuncture vs. Voice Training, one month after treatment) | | | | | | | | | | | |
| 80 (1 RCT) | serious | not serious | not serious | serious | none | ⨁⨁◯◯ Low | 40 | 40 | - | The mean reduction on scores of VHI (Acupuncture vs. Voice Training, one month after treatment) was 0 | WMD 5.67 lower (9 lower to 2.34 lower) |
| Reduction on scores of VHI (Acupuncture vs. Voice Training, two months after treatment) | | | | | | | | | | | |
| 80 (1 RCT) | serious | not serious | not serious | serious | none | ⨁⨁◯◯ Low | 40 | 40 | - | The mean reduction on scores of VHI (Acupuncture vs. Voice Training, two months after treatment) was 0 | WMD 3.75 lower (9.14 lower to 1.64 higher) |

CI: Confidence interval; RR: Risk ratio; WMD: Weighted mean difference; GRB: A tool for evaluation of hoarseness, including Grade (overall grade of hoarseness), Roughness (roughness of voice), and Breathiness (breathiness of voice) by doctors; VHI: Voice handicap indexo.
