# Supplementary material for: Efficacy and safety of acupuncture for vocal nodules: A systematic review and meta-analysis with trial sequential analysis
Source: PLoS One. 2023 Nov 3;18(11):e0288252. doi: 10.1371/journal.pone.0288252 (PMC10624316; doi:10.1371/journal.pone.0288252)
Supplement: S1 File — (PDF) [file pone.0288252.s023.pdf]

Pubmed:

((acupuncture[Title/Abstract]) OR (needle[Title/Abstract]) OR (electro-acupuncture[Title/Abstract]) OR (electropuncture[Title/Abstract]) OR (electroacupuncture[Title/Abstract]) OR (acusector[Title/Abstract]) OR (acupoint[Title/Abstract])) AND ((vocal nodule[Title/Abstract]) OR (vocal cord[Title/Abstract]) OR (larynx[Title/Abstract]) OR (laryngeal[Title/Abstract]) OR (vocal cords nodule[Title/Abstract]) OR (hoarseness[Title/Abstract]) OR (speech disorder[Title/Abstract]) OR (voice disorder[Title/Abstract]) OR (voice[Title/Abstract]) OR (vocal[Title/Abstract]) OR (speech[Title/Abstract]))

Embase:

((acupuncture:ab,ti) OR (needle:ab,ti) OR (electro-acupuncture:ab,ti) OR (electropuncture:ab,ti) OR (electroacupuncture:ab,ti) OR (acusector:ab,ti) OR (acupoint:ab,ti)) AND ((vocal nodule:ab,ti) OR (vocal cord:ab,ti) OR (larynx:ab,ti) OR (laryngeal:ab,ti) OR (vocal cords nodule:ab,ti) OR (hoarseness:ab,ti) OR (speech disorder:ab,ti) OR (voice disorder:ab,ti) OR (voice:ab,ti) OR (vocal:ab,ti) OR (speech:ab,ti))
